# Why do mothers never stop grieving for their deceased children? Enduring alterations of brain connectivity and function

**DOI:** 10.3389/fnhum.2022.925242

**Published:** 2022-09-02

**Authors:** Sarah M. Kark, Joren G. Adams, Mithra Sathishkumar, Steven J. Granger, Liv McMillan, Tallie Z. Baram, Michael A. Yassa

**Affiliations:** ^1^Center for the Neurobiology of Learning and Memory, University of California, Irvine, Irvine, CA, United States; ^2^Department of Neurobiology and Behavior, University of California, Irvine, Irvine, CA, United States; ^3^Department of Pediatrics, University of California, Irvine, Irvine, CA, United States; ^4^Department of Anatomy and Neurobiology, University of California, Irvine, Irvine, CA, United States

**Keywords:** maternal grief, child loss, functional connectivity, MRI, salience network neuropsychology

## Abstract

A child’s death is a profound loss for mothers and affects hundreds of thousands of women. Mothers report inconsolable and progressive grief that is distinct from depression and impacts daily emotions and functions. The brain mechanisms responsible for this relatively common and profound mental health problem are unclear, hampering its clinical recognition and care. In an initial exploration of this condition, we used resting state functional MRI (fMRI) scans to examine functional connectivity in key circuits, and task-based fMRI to examine brain network activity in grieving mothers in response to pictures of their deceased child and as well as recognizable deceased celebrities and unfamiliar individuals. We compared nine mothers who had lost an adult child and aged-matched control mothers with a living child of a similar age. Additionally, we collected diffusion imaging scans to probe structural connectivity and complemented the imaging studies with neuropsychological assessments. Increased functional activation in Ventral Attention/Salience Networks accompanied by a reduced activation in the medial prefrontal cortex in response to the deceased child’s picture robustly distinguished the grieving mothers from controls. Heightened resting-state functional connectivity between the paraventricular thalamic nucleus (PVT) and the amygdala distinguished the grieving mothers from the controls and correlated with subjective grief severity. Structurally, maternal grief and its severity were associated with alterations in corticolimbic white matter tracts. Finally, grieving mothers performed worse than controls on neuropsychological tests of learning, memory, and executive function, linked with grief severity. Reduced activation in cortical regions inhibiting emotions and changes in the PVT circuitry—a region involved in long-term emotional memories and decision making under conflict—distinguish grieving mothers from controls. Notably, the magnitude of neurobiological changes correlates with the subjective severity of grief. Together, these new discoveries delineate a prevalent and under-recognized mental health syndrome and chart a path for its appreciation and care.

## Introduction

Maternal grief over the loss of her child has long been recognized as unique and profound. Grieving mothers are depicted in Greek mythology, The Bible, and works of art. Loss of children to disease, accidents, war, addiction, and suicide affects hundreds of thousands of mothers in the United States and likely millions throughout the world. Remarkably, there is little published research devoted to maternal grief, its origin, brain mechanism and outcomes. Mothers experiencing enduring grief years and decades after their loss are often diagnosed as depressed, hypochondriac or withdrawn, with limited support within and outside of their families. Indeed, the lack of understanding and recognition of prolonged maternal grief hampers families, counselors, and physicians, as well as the mothers themselves. The current study assesses the alterations of brain structure and function that underlie prolonged maternal grief, with the hope of delineating clear neurobiological mechanisms of this important, understudied entity.

The loss of a child violates accepted norms that associate death with older age and severs the strongest human bond- that of a mother and child (Kersting et al., 2009). Losing a child—as opposed to a parent, spouse, or sibling—is the strongest predictor of developing enduring grief (Gündel et al., 2003). Following the loss of any loved one, about 10% of individuals develop enduring grief; in contrast, up to 94% of parents carry enduring grief for their lost child for the rest of their lives. Mothers who have lost a child are particularly vulnerable to enduring grief regardless of how many years have passed. Whereas, prior research focused on the loss of young children, infants, and unborn children, the loss of an adult child, has received limited attention (Zetumer et al., 2015; Arizmendi et al., 2016; McConnell et al., 2018) and is yet to be investigated.

An important question is whether maternal grief is simply a profound, extreme grief or, alternatively, is unique and distinct because of its additional engagement of intrinsic, evolutionarily conserved circuits involved in motherhood together with the emotional circuits more generally underlying grief. In support of the latter hypothesis, grieving mothers themselves report that their grief for their children is different from any other emotion, including grief for other family members. They report the feeling that they have lost a part of their body, of their being. They also distinguish their behaviors in response to the numerous cues related to their children, which provoke uncontrollable crying. They report that the associated tears differ from those provoked by pain or other types of grief. Importantly, grieving mothers distinguish their grief from depression, indicating that they can be appropriately happy on festive occasions, yet their grief occupies a major portion of their existence. This distinction is supported by empirical evidence that grief is a separable construct from depression and post-traumatic stress disorder (Gündel et al., 2003; Arizmendi et al., 2016). Whereas, the observations above support the notion that maternal grief for the loss of a child is a discrete entity with distinguishable neurobiological underpinnings, there is little empirical evidence available for these underpinnings, and the extant data tend to focus on the loss of an unborn child (Zetumer et al., 2015) or the death of a first degree relative or close friend (Shear et al., 2006; Newson et al., 2011; Pohlkamp et al., 2019).

Here, we report on experiments aiming to uncover the neurobiological basis of maternal grief following adult child loss. We recruited a cohort of grieving mothers and age-matched control mothers and examined their brain structure and blood-oxygen-level-dependent (BOLD) functional magnetic resonance imaging (fMRI) brain responses at rest and to cues that involved viewing familiar and unfamiliar faces as well as images of their own deceased children. We hypothesized that responses to photos of the deceased child photo would be aberrantly augmented throughout the salience/ventral attention network in grieving mothers compared to controls. We also hypothesized that, consistent with observations of uncontrollable crying, there would be reduced frontal cortical modulation suggesting impaired inhibition in this network. With respect to resting state fMRI, we specifically tested the hypothesis that grieving mothers would have elevated connectivity of the amygdala to the paraventricular thalamic nucleus (PVT), a region that modulates responses to long-term salient or emotional memories, especially under conflict (e.g., approach/avoidance). The latter features prominently in grieving mothers’ emotional reactions to memories of their children (conflict between the desire to connect with and remember the deceased child and the urge to avoid painful reminders). We also tested the hypothesis that corticolimbic white matter pathways would be altered in grieving mothers compared to controls. Finally, we assessed learning, memory and executive function performance across groups and tested the hypothesis that grief is associated with cognitive deficits in these domains.

## Materials and methods

### Participants and procedures

Between March 2017 and May 2019, we enrolled eighteen mothers, aged 46–72, each with at least 12 years of education (Table 1). Eligible participants were fluent in English with adequate visual and auditory acuity for testing and no history of significant neurologic disease, MRI contraindications, or medical frailty. The Enduring Maternal Grief group (Grief *N* = 9) comprised mothers who lost an adult child unexpectedly (e.g., accident, suicide) at least 5 months before enrollment (median time since loss = 38 months, SD = 110.41, range: 5–362). Grieving mothers were referred through a local support group or by other participants. The control group (Control; *N* = 9) were recruited via community flyers and comprised age-matched women who were parents of a living child close in age to their matched grieving mother’s child at the time of their loss. Controls had never experienced the loss of a child.

**TABLE 1:** Demographic, neuropsychological, and self-report results.

	Grief (*n* = 9*)	Control (*n* = 9*)	Welch’s *t-*test	95% CIs	Hedges’ *g*
**Demographics**					
Age	61.846 (6.109)	57.36 (6.425)	*t*_(15.96)_ = 1.518, *p* = 0.149	[−1.781, 10.752]	0.681
Years of education	14.222 (2.728)	16.889 (2.848)	*t*_(15.971)_ = 2.028, *p* = 0.06	[−5.454, 0.121]	0.911
**Self-report psychological and sleep measures**					
Beck depression inventory *(*n* = 8,9)	17.25 (11.548)	3.556 (3.087)	*t*_(7.889)_ = 3.252, *p* = 0.012	[3.961, 23.428]	1.538
Beck anxiety inventory *(*n* = 8,9)	10.75 (6.756)	2.667 (2.062)	*t*_(8.158)_ = 3.252, *p* = 0.011	[2.371, 13.796]	1.536
Epworth sleepiness scale *(*n* = 7,9)	6.143 (3.761)	6.778 (2.774)	*t*_(10.713)_ = 0.374, *p* = 0.715	[−4.379, 3.109]	0.182
Pittsburgh sleep quality index *(*n* = 7,9)	4.714 (3.039)	6.111 (2.977)	*t*_(12.905)_ = 0.92, *p* = 0.374	[−4.679, 1.885]	–0.439
**Neuropsychological tests**					
Mini mental state exam	28.667 (1.118)	29 (1.118)	*t*_(16)_ = 0.632, *p* = 0.536	[−1.451, 0.784]	0.284
**Digit span**					
Forward	10.556 (1.878)	10 (2.398)	*t*_(15.132)_ = 0.547, *p* = 0.592	[−1.607, 2.718]	0.246
Backward	6.889 (2.088)	7.889 (3.296)	*t*_(13.533)_ = 0.769, *p* = 0.455	[−3.798, 1.798]	–0.345
**Fluency**					
Phonetic (FAS)	41.444 (14.284)	48.222 (14.202)	*t*_(15.999)_ = 1.009, *p* = 0.328	[−21.011, 7.456]	0.453
Category (Animals)	19.333 (3.64)	21.778 (6.888)	*t*_(12.145)_ = 0.941, *p* = 0.365	[−8.095, 3.206]	0.423
**Trail making test**					
Trails A (s)	26.923 (7.307)	21.189 (4.589)	*t*_(13.462)_ = 1.994, *p* = 0.067	[−0.458, 11.927]	0.895
Trails B (s)	73.977 (31.314)	48.59 (10.936)	*t*_(9.923)_ = 2.296, *p* = 0.045	[0.726, 50.047]	1.031
Trails B-A (s)	47.053 (27.737)	27.401 (9.946)	*t*_(10.024)_ = 2.001, *p* = 0.073	[−2.226, 41.53]	0.898
**RAVLT**					
Last learning trial (A5)	10.667 (2.062)	13.889 (1.054)	*t*_(11.915)_ = 4.175, *p* = 0.001	[−4.905, −1.539]	1.874
Learning over trials (LOT)	2.956 (1.363)	4.178 (1.44)	*t*_(15.952)_ = 1.849, *p* = 0.083	[−2.624, 0.179]	0.83
Immediate recall (A6)	7.778 (3.073)	12.556 (1.236)	*t*_(10.522)_ = 4.327, *p* = 0.001	[−7.222, −2.334]	1.942
Delayed recall (A7)	7.778 (3.153)	12.444 (1.424)	*t*_(11.132)_ = 4.046, *p* = 0.002	[−7.202, −2.132]	1.816
Short term % retention (A6/A5)	0.73 (0.223)	0.906 (0.088)	*t*_(10.442)_ = 2.201, *p* = 0.051	[−0.353, 0.001]	0.988
Long term % retention (A7/A5)	0.715 (0.213)	0.898 (0.106)	*t*_(11.738)_ = 2.31, *p* = 0.04	[−0.357, −0.01]	1.037
Recognition correct	13.333 (1.581)	14.222 (0.833)	*t*_(12.126)_ = 1.492, *p* = 0.161	[−2.185, 0.408]	0.67
Recognition FAs	4.889 (3.855)	0.333 (0.5)	*t*_(8.269)_ = 3.516, *p* = 0.007	[1.584, 7.527]	1.578

Sub-samples with self-report psychological and sleep measures denoted with an asterisks *(*n* = Grief subjects, *n* = Control subjects).

All participants underwent a medical screening interview conducted over the phone to check for MRI contraindications and conditions that would affect study eligibility. To meet the eligibility criteria, all participants had to be fluent in speaking and understanding English, have adequate visual and auditory acuity for neuropsychological testing, be in good general health with no diseases expected to interfere with the study, provide pictures of their child that can be shared with the research team for the fMRI task, able to participate for the duration of the study and in all procedures.

Participants were excluded if they displayed any of the following: significant neurologic disease such as Parkinson’s disease, epilepsy, multiple sclerosis, migraine headaches, or brain cyst, tumor or aneurysm; preexisting disability that is so severe that it precludes assessment of dementia (e.g., severe/profound cognitive impairment with multiple disabilities); MRI contraindications, e.g., pacemakers, aneurysm clips, artificial heart valves, ear implants, metal fragments or foreign objects in the eyes, skin or body; Females who are pregnant or trying to get pregnant.

All participants provided written informed consent in accordance with the University of California at Irvine Institutional Review Board and were compensated for their participation. Participants completed a neuropsychological testing battery followed by questionnaires reporting on their grief symptoms. Participants were then given a 30-min break followed by a 1.5-h MRI scanning protocol.

### Image acquisition

The MRI session began with acquisition of a structural MRI followed by three runs of the fMRI task, two resting-state scans, and one DTI scan. MRI data were acquired on a 3.0 Tesla Philips Achieva scanner, equipped with a 32-channel sensitivity-encoding (SENSE) head coil at the Neuroscience Imaging Center at the University of California, Irvine. First, a high-resolution three-dimensional (3D) magnetization-prepared rapid-gradient echo (MP-RAGE) structural scan was acquired for co-registration (0.75 mm^3^, TR/TE = 11/4.4 ms, 200 slices, FOV = 240 × 231 × 105, flip angle = 18 degrees, slice orientation = sagittal). This was followed by task and resting state fMRI (2.5 mm × 2.5 mm in-plane resolution, 2.3 mm slice thickness with a 0.2 gap, TR/TE = 2,500/26 ms, 44 slices, FOV = 180, flip angle = 70 degrees, slice orientation = transverse, 147 dynamic scans per run, number of dummy scans to ensure T1 stabilization = 4). Diffusion data were acquired with a b-value of 800 s/mm^2^, 32 non-collinear directions and a single volume with a b-value of 0 s/mm^2^. (TR/TE = 1,298/49 ms, FOV = 128, 128, 60 mm, voxel size = 1.78 × 1.78 × 2 mm) (Table 2).

**TABLE 2:** Brain areas that show a group difference on the task fMRI child response (Child > Celebrity + Random) group comparisons.

		MNI coordinates				
Regions	BA	x	y	z	Cluster size	*Z*-score
**Grief > Controls**						
**Inferior parietal lobule**						
Superior temporal gyrus	22	−48	−44	20	469	5.34
Inferior parietal lobule, supramarginal gyrus	40	−58	−50	26	−	4.2
Inferior parietal lobule, supramarginal gyrus	40	−56	−42	26	−	3.86
**Anterior cingulate**						
Mid and anterior cingulate cortex	32	−4	20	40	444	4.78
Superior frontal gyrus	6	−14	10	60	−	4.31
Midcingulate, dorsomedial frontal cortex, supplementary motor area	6	4	10	46	−	4
**Middle frontal gyrus**	9	−30	44	36	50	4.23
**Insula (right)**						
Inferior orbital frontal gyrus	47	52	16	−4	197	4.19
Anterior insula	13	44	10	0	−	4
Superior temporal pole	38	56	12	−12	−	3.81
**Visual cortex**						
Superior occipital gyrus	19	−16	−84	16	334	4.08
Cuneus, Calcarine sulcus	17, 18	6	−80	28	−	4.04
Cuneus	18	6	−86	38	−	3.96
**Insula (left)**						
Anterior insula	13	−40	10	6	81	4.08
Subcentral gyrus	4	−56	0	8	−	3.34
Insula, subcentral gyrus	4	−46	0	8	−	3.02
**Middle frontal gyrus**		52	16	42	60	4.06
**Precuneus, superior parietal lobule**	7	−12	−72	46	63	3.91
**Occipito-parietal cortex**						
Superior occipital gyrus	19	−30	−60	26	194	3.76
Inferior parietal lobule, angular gyrus	39	−36	−60	40	−	3.45
Cuneus	23	−20	−52	28	−	3.33
**Cerebellum**						
Cerebellum crus I	−	48	−66	−30	145	3.61
Cerebellum crus I	−	30	−64	−30	−	3.47
Cerebellum crus I	−	40	−52	−32	−	3.18
**Precuneus**	7	−4	−72	52	54	3.58
**Cuneus**	19	−16	−84	36	51	3.58
**Inferior frontal gyrus, pars triangularis**	45	−40	38	26	50	3.41
**Inferior frontal gyrus, pars triangularis**	45	−46	38	18	−	3.14
**Brainstem**	−	−2	−24	−28	56	3.39
**Controls > Grief**						
**Lateral occipital cortex**						
Middle occipital gyrus		−54	−74	16	76	3.69
Middle occipital gyrus		−50	−82	10	−	2.72
**Precuneus add**		8	−50	40	55	3.57

Results are corrected for multiple comparisons using a cluster size threshold of 49 voxels.

### Task-activated functional magnetic resonance imaging analysis

**FIGURE 1:**
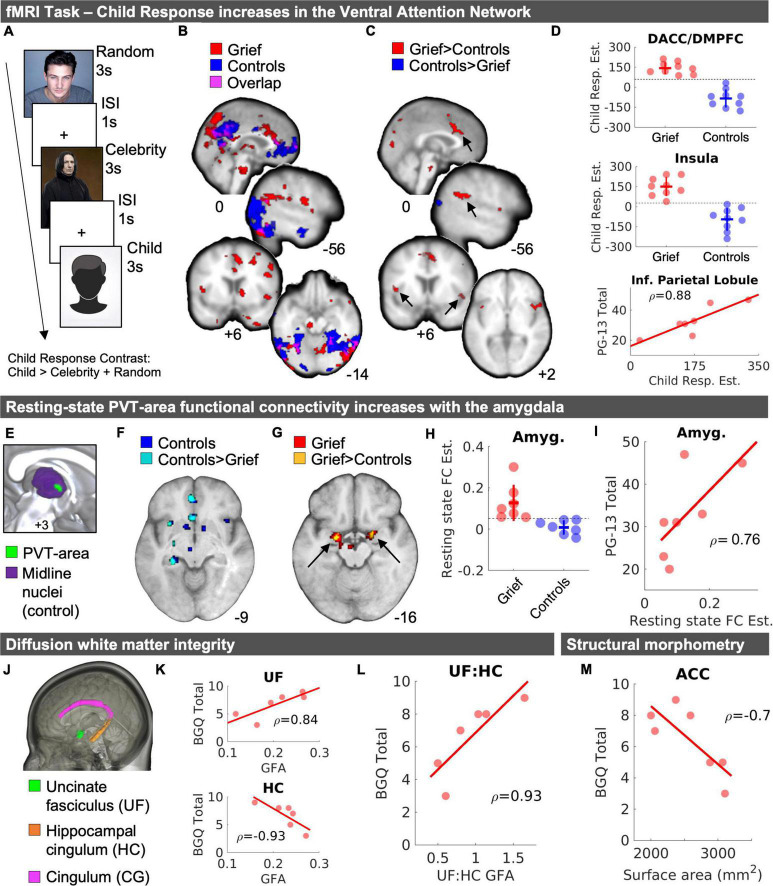
MRI results. **(A)** Task structure. **(B)** Child fMRI Response (Child > Celebrity + Random) for Grief (red) and Control (blue) mothers and the spatial overlap of voxels shared between groups (violet). **(C)** Independent samples *t-*test results demarcate greater Child fMRI Responses in Grief compared to Controls (red) and Controls compared to Grief (blue). Arrows point to regions with corresponding plots in panel **(D)**. **(D)** Individual data points of Child fMRI Response magnitude in the dACC/DMPFC (top) and insula (middle) shown with standard deviation crossbars and dotted line illustrates numerical split between groups. The bottom panel of part **(D)** depicts a correlation between Child fMRI Response magnitude and subjective grief on the PG-13 in the inferior parietal lobule. **(E)** Midline view of PVT-area resting state functional connectivity analysis seed regions. **(F)** Resting-state fMRI results of PVT-area functional connectivity in controls (blue) and voxels that showed greater connectivity in controls compared to Grief mothers (cyan). **(G)** Resting-state fMRI results of PVT-area functional connectivity in Grief mothers (red) and voxels in the amygdala that showed greater connectivity in Grief mothers compared to controls (yellow). **(H)** Individual datapoints of PVT-area functional connectivity by group. The horizontal line highlights the complete numerical split between the groups. **(I)** Follow-up correlation analysis of PVT-area functional connectivity and PG-13 subjective grief scores. **(J)** Rendering of areas of interest in the DTI analysis. **(K)** Correlation plots between BGQ Total and UF (top) and HC (bottom). **(L)** Correlation plot shown for BGQ Total and UF: HC GFA ratio. Spearman’s rho (ρ) values are displayed with correlations. **(M)** Correlation between ACC surface area and subjective grief scores on the BGQ Total. DACC, dorsal anterior cingulate cortex; DMPFC, dorsomedial prefrontal cortex; PVT, paraventricular nucleus of the thalamus; Amyg., amygdala; FC, functional connectivity; PG-13, prolonged grief questionnaire; ACC, anterior cingulate cortex; GFA, generalized fractional anisotropy; BGQ, brief grief questionnaire.

Upon enrollment in the study, participants provided 30 images of their child. During the fMRI task, the 30 images of their child were presented randomly intermixed with 30 images of recently deceased celebrities (familiarity control condition) and 30 images of unfamiliar individuals from stock photos (unfamiliar control condition). The photos of their child could be from across the lifespan at any age, subjects typically provided a breadth, but no strict divisions were made. The main stipulation was that the child needed to be front facing and the only person in the photo. Images were either provided digitally, or physical copies were scanned. Appropriate cropping and other small edits were made to ensure photos fit within the criteria for the task. Each of the 90 images was shown three times for 3 s, with a 1-s fixation image between trials (Figure 1A). Participants were asked to rate their emotional response to each image on a scale of 1 to 4 using an MR compatible button box.

Pre-processing and subject-level general linear model analyses were implemented using FSL FEAT. Each subject’s individual images were motion-corrected, smoothed with a 5 mm Gaussian filter, high-pass filtered, and pre-whitened. Event-related responses were estimated using event-related convolution with an ideal hemodynamic response represented by a double gamma function and its temporal derivative. Functional images were registered to their corresponding T1-weighted anatomical image and then to the standard MNI152 2 mm template. Explanatory variables were modeled for each of the three conditions (Child, Celebrity, Random). The Child Response contrast, i.e., Child > [Celebrity + Random], for each subject was entered into a group-level independent samples *t*-test in SPM12 (Wellcome Department of Cognitive Neurology, London, United Kingdom). In addition, the Child Response subject-level contrast images were entered into correlational models to demarcate clusters with positive and negative correlations between the fMRI Child Response magnitude and the subjective-grief scores. Task fMRI statistical maps were set to an initial voxel threshold of *p* < 0.005 (uncorrected). The group comparison and subjective grief correlation result maps were corrected for multiple comparisons to *p* < 0.05 by enforcing a cluster-forming voxel extent threshold of k = 49 based on Monte Carlo Simulations with 1,000 iterations (Slotnick, 2017). The group comparison contrast (effectively a directional interaction) was inclusively masked with the corresponding group map thresholded at *p* < 0.005.

### Resting state functional magnetic resonance imaging analysis

Resting-state volumes were preprocessed and successfully denoised using the CONN Toolbox (Whitfield-Gabrieli and Nieto-Castanon, 2012) version 19c. The structural images were centered, AC-PC aligned, segmented, and normalized to the MNI template. The functional images were (Kersting et al., 2009) realigned and unwarped, (Gündel et al., 2003) centered and aligned to the AC-PC line, (McConnell et al., 2018) slice-timing corrected, (Arizmendi et al., 2016) segmented and MNI-normalized, and (Zetumer et al., 2015) smoothed with a 6 mm Gaussian kernel. Structural and functional data were resampled to 1 and 2 mm^3^ voxels, respectively. The pre-processed images underwent despiking followed by nuisance regression (white matter and CSF signal using aCompCor, realignment with first order derivatives, framewise motion estimates, motion artifact scrubbing, and linear detrending) followed by band-pass filtering (0.008 Hz < f < 0.09 Hz). We enforced conservative motion artifact thresholds (Global Signal Change z > 3 and framewise displacement > 0.5 mm). Two Grief participants (aged 54 and 55) and 1 control (age 50.95) were excluded from RSFC analyses for having far fewer valid scans than the rest of participants (more than 30% of the timepoints scrubbed) and instances of max motion > 4 mm (Supplementary Figure 1). Upon removing these three participants from analysis, denoising was successful with no evidence of associations between random permutations of RSFC and the quality control (QC) metrics (valid number of scans, mean or max global signal change or mean motion). All included participants had approximately 12 min of usable data (Controls: Mvalidscans = 292.6; Grief: Mvalidscans = 291.3) and there was no evidence that the number of valid scans or other motion metrics differed between groups.

We focused on delineating the circuitry of paraventricular thalamic nucleus (PVT), in view of its established and emerging roles in the encoding and integration of emotionally salient long-term memories (Do Monte et al., 2016; Kark et al., 2021; Kooiker et al., 2021) and the influence of these memories on behaviors during emotional conflicts (Choi et al., 2019). We assessed the impact of maternal grief on resting-state functional connectivity (RSFC) of the PVT. The PVT-area seed was created using the left and right Pv.nii.gz files received in 0.5 mm MNI-space (Krauth et al., 2010; Jakab et al., 2012). We first combined the left and right PVT seeds from the atlas using imcalc in SPM12 and then slightly dilated the bilateral PVT seed using MRICRON (Dilate drawing feature^1^). In CONN, all seed regions were inclusively masked with subject-level structural gray matter segmentation masks to ensure signal extraction from gray matter. For one grief participant with a wider third ventricle width, the PVT seeds were slightly nudged away from x = 0 using MRICRON to fall on the paraventricular gray matter. This approach was based on our recently published work demonstrating the intrinsic structure and connectivity of the PVT in humans (Kark et al., 2021). Given the small size of the PVT and to address potential confounding effects of neighboring nuclei, we created a midline subnuclei control seed encompassing all midline nuclei using the Thalamus Atlas of the Swiss Federal Institute of Technology (ETH) in Zurich and University of Zurich, Switzerland (Krauth et al., 2010; Jakab et al., 2012). Subject-level seed-to-voxel PVT RSFC maps were calculated, controlling for the midline subnuclei, and subsequently entered a group-level independent samples *t*-test in SPM12. Group-level analyses were spatially constrained to key nodes of PVT functional connectivity (Kark et al., 2021; Kooiker et al., 2021), which include the orbitofrontal cortex, cingulate cortex, amygdala, hippocampus, striatum, and the insula, to increase the sensitivity of the analysis and thresholded at *p* < 0.05 uncorrected.

### Diffusion weighted imaging analysis

As the potential impact of maternal grief could involve altered structural connectivity of salient nodes in several “emotional” circuit, we assessed the integrity of several white matter tract ROIs including the cingulate gyrus (CG), hippocampal cingulum (HC), and uncinate fasciculus (UF) (Figure 1J). Like our prior work (Granger et al., 2021), data were analyzed using DSI-Studio,^2^ corrected for motion using FSL’s *eddy_correct* algorithm, and reconstructed using DSI Studio Q-spin Diffeomorphic Reconstruction (QSDR) algorithm which computes orientation distribution functions in MNI space (Yeh and Tseng, 2011). White matter tract integrity was measured by extracting generalized fractional anisotropy (GFA) from each of the three ROIs (obtained from the Johns Hopkins white matter atlas in DSI studio) and averaging across hemisphere. One Grief subject (age 58) was excluded from the GFA analyses because of a suboptimal template fit (R^2^ value of 0.63), as recommended by the software developers. Like our previous work, corticolimbic circuitry imbalance was calculated as the ratio of UF GFA: HC GFA (Granger et al., 2021).

### Structural morphometry

Using the Freesurfer 6.0 and the DKT atlas (Fischl et al., 2002, 2004), we quantified cortical surface areas of seven regions of interest (e.g., cingulate, prefrontal regions) and subcortical volumes of seven regions of interest (e.g., striatal regions, amygdala, hippocampus). An affine registration with Talairach space is followed by an initial volumetric labeling and correction for variation in intensity due to the B1 bias field. After this, a high dimensional non-linear volumetric alignment to the Talairach atlas was performed, followed by pre-processing, and finally the volume was labeled. Combining across hemisphere, we assessed subcortical volumes (accumbens_area, Amygdala, caudate, putamen, amygdala, hippocampus, Thalamus_ Proper) and surface areas (entorhinal_area, parahippocampal_ area, insula_area, anterior cingulate [caudalanteriorcingulate_ area+rostralanteriorcingulate_area], medialorbitofrontal_area, ventro-lateral PFC [parsopercularis_area+parsorbitalis_area+ parstriangularis_area], and middle-frontal/DLPFC areas [ros tralmiddlefrontal_area+caudalmiddlefrontal_area]). Volumes were reported as a percentage of the total gray matter volume and surface areas are reported in mm^2^.

### Neuropsychological and self-report measures

Participants completed a battery of tests including the Mini Mental State Exam [MMSE] (Folstein et al., 1975); the Wechsler Digit Span Forward and Backward, The Controlled Oral Word Association Test (COWAT), Semantic Verbal Fluency (Animals), Trail Making Test A and B, and the Rey Auditory Verbal Learning Test (Schmidt, 1996). They also completed self-report assessments of depressive symptoms (Beck Depression Inventory–II [BDI]) (Beck et al., 1996), anxiety-related symptoms (Beck Anxiety Inventory [BAI]) (Beck and Steer, 1993), sleep quality [Epworth Sleepiness Scale] (Johns, 1991) and Pittsburgh Sleep Quality Index [PSQI] (Buysse et al., 1989). Additionally, the Grief group completed two subjective grief assessments: The Prolonged Grief Disorder-13 scale (PG-13) (Prigerson et al., 2009) and the Brief Grief Questionnaire (BGQ) (Shear and Essock, 2002).

The PG-13 is a diagnostic tool (Prigerson et al., 2009) to assess if respondents meet criteria for Prolonged Grief Disorder (PGD). The PG-13 considers PGD experiencing daily separation distress for at least 6 months post-loss, daily or pervasive cognitive, emotional, and behavioral symptoms, and self-reported impairment in functional areas of living (e.g., social, occupational, domestic responsibilities).

The BGQ is a reliable and valid tool for assessing subjective grief (Shear and Essock, 2002). The BGQ asks responders to rate the extent to which they feel cut off or distant, avoid things related to the person who died, experience bothersome thoughts or images, have trouble accepting the death, and how much grief still interferes with their life (0 = not at all, 1 = somewhat, 2 = a lot). On the BGQ, a score of 8 or higher indicates likely complicated grief, scores ranging from 5 to 7 are considered subthreshold, and a score less than 5 is considered unlikely complicated grief.

### Data analysis and visualization

Our general approach was to first test hypothesized regions and circuits in group-level *t*-tests. For all group comparisons, we calculated independent samples *t*-test statistics (assuming unequal variances) and Hedge’s *g*. We subsequently tested across-subject correlations within the Grief group using Spearman’s rho (ρ). The 3D statistical brain maps were visualized for figures using MRIcroGL^3^ and MRICRON.^4^ Group comparison plots were created using beeswarm.m.^5^

## Results

### Heightened salience network responses to picture of the deceased child

In-scanner emotionality ratings were entered into a 2 × 3 repeated measures ANOVA with the between subject factors of group (Grief, Controls) and the within-subject factor of stimulus type (random, celebrity, child). There was evidence for a stimulus by group interaction [*F*_(2,32)_ = 6.42, *p* = 0.005]. Both groups of mothers rated viewing images of their children with similar levels of emotionality (*M* = 3.92, SE = 0.20) but the Grief mothers rated the images of deceased celebrities as relatively less emotional compared to the control mothers (*t*_(10.27)_ = 3.98, *p* = 0.002, Hedges’ *g* = 1.79 [0.56 2.96]). The in-scanner emotion ratings for images of their child were at ceiling for both groups, thus we were not able to parametric modulation of the Child Response by emotionality rating.

In both grieving and control mothers, the viewing images of their children was associated with activation of mPFC, precuneus, lateral occipital cortex, and the ventral visual stream (Figure 1B, violet). In the Grief group, activation of anterior and posterior cingulate was noted. Unexpectedly, the dorsomedial thalamus including the paraventricular thalamic nucleus (PVT) was robust (Figure 1B, red), as well as brain stem regions. Notably, activation of the PFC robustly distinguished the groups: In the Control group only, mPFC activation was spatially expansive and extended anteriorly toward the frontal pole, and additionally involved the lateral PFC, orbitofrontal cortex and middle temporal gyrus (Figure 1B, blue).

When comparing groups directly, we observed that, compared to controls, activation in grieving mothers was reduced in the mPFC (MNI_*xyz*_ = −10, 54, 0; *k* = 19) at a reduced voxel extent threshold minimum, as well as in the precuneus and lateral occipital cortex (Figure 1C, blue). Conversely, activation was increased in grieving mothers within dorsal anterior cingulate (dACC), dorsal medial prefrontal cortex (dmPFC), anterior insula (AI), medial portions of the visual cortex, and inferior parietal lobule (IPL) (Figures 1C,D). Of these the IPL correlated positively with assessment of severity of the grief (PG-13 scores, Figure 1D, bottom). At a reduced voxel extent threshold minimum (*k* = 10) for Grief > Controls, clusters were also observed in the striatum (MNI_*xyz*_ = 16, 2, 2; *k* = 27), including the caudate and putamen, and the midbrain (MNI_*xyz*_ = -2, -24, -28; *k* = 56), including the substantia nigra/ventral tegmental area (SN/VTA).

### Heightened resting state functional connectivity of the paraventricular thalamic nucleus circuit

We assessed the resting state functional connectivity (RSFC) of the PVT structure in the Grief group compared with controls. In the controls, one-sample *t*-tests yielded the expected PVT circuitry, reproducing prior work (Kark et al., 2021), including hippocampus, amygdala, nucleus accumbens (NAc), anterior cingulate cortex (ACC), and ventral tegmental area (VTA) (shown in blue in Figure 1F). This first analysis established the ability to detect PVT region RSFC with expected targets in our relatively small sample (Table 3 and Figures 1E–I). In the Grief group, the PVT was functionally connected to amygdala, hippocampus, ACC, and VTA (shown in red in Figure 1G). Examining group differences using independent samples *t*-tests distinguished the Grief and Control groups: Compared to grieving mothers, control mothers showed relatively greater PVT functional connectivity with the ACC, hippocampus, and insula (shown in cyan in Figure 1F). Compared to control mothers, connectivity of PVT with the VTA and bilateral amygdala was greater (shown in yellow in Figure 1G and plotted in Figure 1H). PVT-amygdala functional connectivity of individual grieving mothers correlated with their subjective prolonged grief score (ρ = 0.76) (Figure 1I). Notably, we observed a complete numerical split between the groups in PVT functional connectivity with the amygdala (Figure 1H).

**TABLE 3:** Resting-state paraventricular thalamic nucleus (PVT)-area functional connectivity results (*p* < 0.05 uncorrected).

		MNI coordinates				
Regions	BA	x	y	z	Cluster size	Z-score
**Controls**						
**Hippocampus**	−	−16	−8	−16	77	3.54
Hippocampus	−	−28	−14	−14	−	3.45
Amygdala	−	−20	0	−22	−	1.82
**Amygdala**	−	30	2	−16	19	3.3
**Nucleus accumbens**	−	12	8	−10	10	3.01
**Anterior cingulate cortex**	32	6	40	6	48	2.99
Anterior cingulate cortex	32	4	50	8	−	1.88
Anterior cingulate cortex	32	−4	48	8	−	1.88
**Hippocampus**	−	−24	−32	−10	32	2.99
**Anterior cingulate cortex**	32	−4	42	−10	68	2.94
Anterior cingulate cortex	32	−4	34	−4	−	2.63
Anterior cingulate cortex	32	−6	52	−4	−	1.98
**Ventral tegmental area**	−	−4	−18	−10	13	2.89
**Anterior cingulate cortex**	32	0	20	−8	30	2.79
**Insula**	13	−26	16	−12	19	2.46
**Nucleus accumbens**	−	−8	8	−12	15	2.4
**Insula**	13	40	18	−14	15	2.38
Insula	13	36	16	−6	−	1.92
**Controls > Grief**						
**Hippocampus**		−24	−34	−10	20	3.62
**Amygdala**		30	2	−16	11	3.48
**Anterior cingulate cortex**		6	40	6	14	3.32
**Hippocampus**		−24	−10	−10	15	3.24
**Anterior cingulate cortex**		−4	46	−10	26	3.17
**Insula**		−30	12	−10	17	2.9
**Anterior cingulate cortex**		0	22	−10	23	2.61
**Grief**						
**Amygdala**	−	−18	−6	−14	85	3.89
**Amygdala**	−	20	2	−16	65	3.1
Hippocampus	−	16	−10	−12	−	2.51
**Ventral tegmental area**	−	−4	−14	−12	26	2.93
**Anterior cingulate cortex**	32	−6	50	10	21	2.6
**Anterior cingulate cortex**	32	6	50	6	11	2.39
**Grief > Controls**						
**Amygdala**	−	20	2	−16	17	2.53
**Ventral tegmental area**	−	−4	−14	−12	10	2.48
**Amygdala**	−	−20	−4	−16	16	2.34

### Imbalance in corticolimbic white matter tract integrity

Although DTI scan head motion was greater in the Grief group compared to controls, BGQ Total scores and UF:HC ratios were not related to head motion across subjects (Supplementary Figure 4). Grieving mothers had a lower hippocampal cingulum (HC) GFA [*t*_(11.54)_ = 2.18, *p* = 0.051, CIs (−0.037, 2.037), Hedges’ *g* = 1.02] and uncinate fasciculus (UF) GFA [*t*_(11.675)_ = 1.41, *p* = 0.184, CIs (−0.342, 1.625), Hedges’ g = 0.66] values compared to the controls, but the differences did not reach statistical significance. While the effect sizes were medium-large, it is important to note that sample size is a limitation. There was no pattern toward a GFA difference of the cingulum [*t*_(14.796)_ = 0.73, *p* = 0.48, CIs (1.29, 0.63), Hedges’ g = 0.34]. Of note, higher levels of subjective grief (BGQ Total) were associated with reduced HC-GFA (Figure 1K, top), greater UF-GFA (Figure 1K, bottom), and thus an increase ratio of UF/HC GFA (Figure 1L).

### No major structural magnetic resonance imaging differences between groups

Whereas significant differences in functional connectivity and long-range corticolimbic white matter tracts distinguished grieving mothers and controls, we did not detect group differences in volume or surface areas of the tested brain regions (Supplementary Figures 2, 3). Higher levels of subjective grief were associated with reduced cortical surface area over the ACC (Figure 1M).

### Impaired learning efficiency, verbal memory, and executive function

**FIGURE 2:**
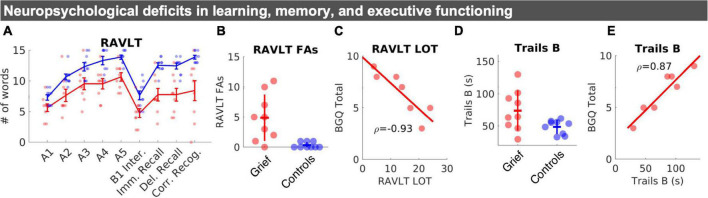
Neuropsychological changes in learning, memory, and executive functioning. **(A)** Mean RAVLT word list scores by group: Learning trials (A1–A5), interference list (B1), immediate and delayed recall, and corrected recognition (# of correct hits–# of incorrect false alarms). Standard error bars and individual datapoints are shown. **(B)** Individual datapoints for RAVLT false alarms shown with SD crossbars. **(C)** Correlation between BGQ and learning efficiency (LOT index five Total of trials (I to V)–(5X [A1])). **(D)** Individual datapoints for Trails B shown with SD crossbars. **(E)** Correlation between Trails B performance, in seconds, and subjective grief on the BGQ. RAVLT, rey auditory verbal learning test; FAs, false alarms; LOT, learning over trials.

The groups did not differ on the MMSE. Tests of working memory or phonemic and semantic verbal fluency also did not differ between groups. In tests of episodic memory, grieving mothers performed worse than controls on multiple measures of the RAVLT across item recall trials (Figure 2A), including the Last Learning Trial (A5), Immediate Recall, Delayed Recall, and an increased number of Recognition False Alarms (Figure 2B), which drove down Corrected Recognition Scores (Hits—FAs) (Figure 2A). Grieving mothers retained fewer items in memory, even when correcting for the number of items learned (i.e., Long Term Retention). Across participants (*N* = 7), higher levels of subjective grief were associated with the Learning Over Trials (LOT) index, a measure of learning efficiency (Figure 2C).

Whereas, group differences in psychomotor performance on Trails A were non-significant, we observed evidence for reduced executive function performance on the Trail Making Test B in the Grief group (Figure 2D). Across participants (*N* = 7), poorer Trails B performance was closely linked with higher levels of subjective grief (Figure 2E). Groups did not differ on self-reported sleep measures. Yet, grieving mothers scored higher than controls on the BDI and BAI (Table 1).

Acknowledging prior work that grief is a unique condition from mood or anxiety disorders, we utilized the diverse items on the BDI-II (cognitive, affective, and somatic symptoms) to further understand symptoms driving BDI-II scores in this sample. Item scores on the BDI-II were compared between the two groups and sorted in descending order based on effect size (Hedge’s *g*). The “difficulty concentrating” item emerged as the most consistent symptom of the Grief group with the largest between-group effect size. Only one of the control mothers indicated mild concentration difficulty, by contrast, all eight of the Grief mothers with BDI-II data reported at least mild (*n* = 4) or moderate (*n* = 4) difficulty concentrating (see heat map of item scores by participant and group in Supplementary Figure 5). All the Grief mothers also reported mild levels of tiredness and fatigue, and anhedonia symptoms appeared elevated in those with higher PG-13 scores (participant columns sorted by PG-13 scores, see x-axis in Supplementary Figure 5). Interestingly, the mothers with the two highest subjective grief scores had no overlap of their moderate symptoms on the BDI-II except for difficulty concentrating.

Of note, grieving mothers who felt overwhelmingly bitter and had trouble accepting loss reported the highest levels of grief. On the BGQ, all seven of the mothers endorsed either “Somewhat” or “A lot” when asked with how much grief interfered with their life and how much they were experiencing bothersome images or thoughts of their child when they died or thoughts about the death. Feeling cut off or distant from people, family, or friends was the least severe symptom (*n* = 3 “Not at all”, *n* = 4 “Somewhat”).

On the PG-13 survey (*n* = 7 total), six Grief mothers reported weekly (*n* = 3) or daily (*n* = 3) intense feelings of emotional pain, sorrows, or pangs of grief. Six reported weekly (*n* = 2) or daily (*n* = 4) yearning or longing for their lost child. Only two reported avoidance of reminders. Six of the seven Grief mothers with the PG-13 data reported a pronounced reduction in functional areas of life (item 13). Of these seven mothers, one also met criteria for prolonged separation distress (criterion based on items 1–3), one met the PG-13 criteria for frequent cognitive, emotional, and behavioral symptoms (items 4–12), and two met criteria for both—indicative of PGD based on the PG-13. Naturally, those two mothers had the highest PG-13 scores (45 and 47), and both reported “overwhelming” feelings of bitterness about their loss (versus the five others who reported “somewhat” bitter or less). There two mothers also indicated trouble accepting the loss and confusion about their role in life since the loss.

Consistent with prior work, there was insufficient evidence for a link between time since loss or age at the time of loss and subjective grief, suggesting subjective grief did not decrease with time. In fact, the mother with the highest subjective grief scores had lost her son about 30 years prior to testing.

## Discussion

This study provides novel neurobiological and neuropsychological correlates of enduring grief in mothers who have lost an adult child. Our results provide evidence of functional, structural, and neurocognitive changes associated with the presence and severity of enduring maternal grief. Four major features characterize grieving mothers: First, cues of the deceased child evoke activation throughout the Ventral Attention and Salience Networks (VAT and SN). Second, compared with controls, the activation in a specific mPFC cluster is attenuated, suggesting reduced cortical inhibition. Third, at rest, functional coupling of the PVT and the bilateral amygdala is heightened. Fourth, there is an imbalance in the white matter integrity of the corticolimbic circuitry—with an aberrantly elevated ratio of uncinate fasciculus to hippocampal cingulum integrity. Lastly, learning efficiency, verbal memory, and executive function are lower in grieving mothers. Together, these findings demonstrate that enduring maternal grief involves a distinct array of neurobiological and neuropsychological systems demonstrating that mothers who have suffered the loss of a child are “Forever Changed” and providing credence to their real and unique “syndrome.”

When shown photos of their child, both groups of mothers similarly activated midline anterior and posterior cingulate, and the precuneus—a pattern consistent with personal familiarity with a close family member (Donix et al., 2010), maternal caregiving (Gholampour et al., 2020), and processing and remembering emotionally meaningful and self-relevant stimuli (Gutchess and Kensinger, 2018). These results indicate the task was successful in engaging the maternal caregiving circuitry and networks associated with self-relevant memories.

Notably, mPFC activation was present in Control mothers and relatively attenuated in Grieving mothers. This finding is consistent with reports from grieving mothers of uncontrollable crying with tears of unique qualities. These reports are consistent with a loss of prefrontal control over subcortical regions, and the generation of a subcortical syndrome characterized by uncontrollable crying or laughing, as described with PFC strokes or neurodegeneration (Ghaffar et al., 2008; Christidi et al., 2018).

Our findings are consistent with prior work that has shown grief impacts function of the anterior cingulate and the insula (Silva et al., 2014; O’Connor, 2019)—regions also involved in maternal caregiving circuitry (Kersting et al., 2009; Stoeckel et al., 2014; Paul et al., 2019; Gholampour et al., 2020) and physical and social pain (Eisenberger, 2006). The dACC and AI are primary nodes of the VAT and SN, which integrate emotional, cognitive, motivational, and homeostatic information to detect and identify biologically relevant stimuli to guide behavior and actions (Menon, 2015). In grief neuroimaging work, activation of the AI is associated with reminders of unattainable rewards (Naqvi et al., 2007), consistent with separation distress in grief (Freed et al., 2009; Silva et al., 2014). While activation of the dACC and AI completely distinguished the Grieving from the Control mothers, the magnitude of these effects was not related to subjective levels of grief. Yet, activation of a node of the VAT—the IPL—correlated with subjective grief.

During the resting state, RSFC between the PVT and both amygdalae—a functional connection associated with emotionally salient long-term memories (Do-Monte et al., 2015; Barson et al., 2020; Fernández-Alcántara et al., 2020; Kooiker et al., 2021) was dramatically heightened. This circuitry has also been associated with arbitration under motivation conflict (Choi et al., 2019), which is salient, because grief can be viewed as a conflict—between simultaneous yearning to connect with the deceased child and avoiding painful reminders. These findings suggest a long-term alteration of PVT circuitry as an intrinsic correlate of child loss grief.

In additional to functional changes, corticolimbic imbalances between the UF and HC white matter GFA were apparent in the Grief group—consistent with recent work that adverse life experience can induce an imbalance in the maturation of these two corticolimbic circuit tracts (Granger et al., 2021). Both the UF and the HC connect the medial temporal lobes with frontal executive circuits, and their imbalance may lead to altered input to the PFC that may adversely impact its executive functions, in accord with the neurocognitive difficulties identified in the grieving mothers. Structurally, increased levels of subjective grief were associated with decreased surface area of the ACC, another important control region involved in processing negative emotions as well as monitoring and resolving conflict (Dignath et al., 2020).

In parallel to and concordance with neuroimaging, child loss grief was associated with selective neurocognitive dysfunction in the domains of learning, memory, and executive function. Prior work has shown that grief can impact global cognitive function (Hall et al., 2014), visuo-spatial and attentional function, and immediate and long-term memory (Pérez et al., 2018). The current neuropsychological findings validate the subjective cognitive complaints expressed by grieving mothers and provide evidence for disruptions in cognition and memory that may contribute to the grief severity.

A limitation of the current work is the small sample size owing to the difficult recruitment in this population and the sensitivity and burden of the testing procedures. Thus, results from this work should be considered preliminary and we hope that this work will inspire future larger studies in mothers who have lost their children. While we focused on a number of planned analyses of neural and neuropsychological measures, we provided a comprehensive list of all of the regions involved and the neuropsychological tests available for the reader’s benefit. We stress that since these analyses were not corrected for multiple comparisons, they should be considered exploratory and should be treated with caution.

The current study provides a critical foundation for future research, societal attitudes, and policy considerations about maternal grief over the loss of a child. Yet, much additional work is required: understanding grief in fathers (Murphy et al., 1999; McNeil et al., 2021), adoptive parents, and parents of single children, as well as how the course of parental grief is influenced by factors such as preparedness for the death (Kovarsky, 1989; Feigelman et al., 2011), culture (Xiu et al., 2017), and race. The current study included predominantly white college-educated participants, yet Black Americans are two to four times more likely to lose a child than White Americans, and their grief may be steeped in additional cultural factors (Umberson et al., 2017). Finally, current bereavement policies offer between zero to fourteen days of protected time to bereave the loss of a child under the age of eighteen. Considering the profound neurobiological and neuro-cognitive processes involved in such grief, as reported here, policymakers and employers need to consider extended bereavement benefits and support for gradual reentry to the workplace. The current work highlights that child loss grief is not temporally constrained and endures for years and decades. We consider our work a first step toward recognition and understanding of the basis and impact of this devastating condition.

## Data availability statement

The original contributions presented in this study are publicly available. This data can be found here: https://osf.io/nhgua/.

## Ethics statement

The studies involving human participants were reviewed and approved by University of California Irvine Institutional Review Board. The patients/participants provided their written informed consent to participate in this study.

## Author contributions

TZB and MY designed the experiments. JA recruited participants and collected the data. SK, JA, MS, and SG analyzed the data. SK wrote the first draft of the manuscript and TZB and MY provided extensive edits. All authors contributed to the article and approved the submitted version.

## Funding

This study was supported by a generous private gift from Irwin and Elaine Weinstein, by the Hewitt Foundation for Biomedical Research (to SK), and NIH grants P50MH096889 (PI: TZB) and R01MH102392 (PI: MY).

## Footnotes

1
http://people.cas.sc.edu/rorden/mricron/index.html

2
http://dsi-studio.labsolver.org

3
http://www.mccauslandcenter.sc.edu/mricrogl/home

4
http://people.cas.sc.edu/rorden/mricron/index.html

5
https://github.com/ihstevenson/beeswarm
