# Supplementary material for: Why do mothers never stop grieving for their deceased children? Enduring alterations of brain connectivity and function
Source: Front Hum Neurosci. 2022 Sep 2;16:925242. doi: 10.3389/fnhum.2022.925242 (PMC9478601; doi:10.3389/fnhum.2022.925242)
Supplement: Supplementary file 1 [file Data_Sheet_1.DOCX]

**Why Do Mothers Never Stop Grieving for Their Deceased Children? Enduring Alterations of Brain Connectivity and Function**

Kark et al.

**SUPPLEMENTARY FIGURES**

**
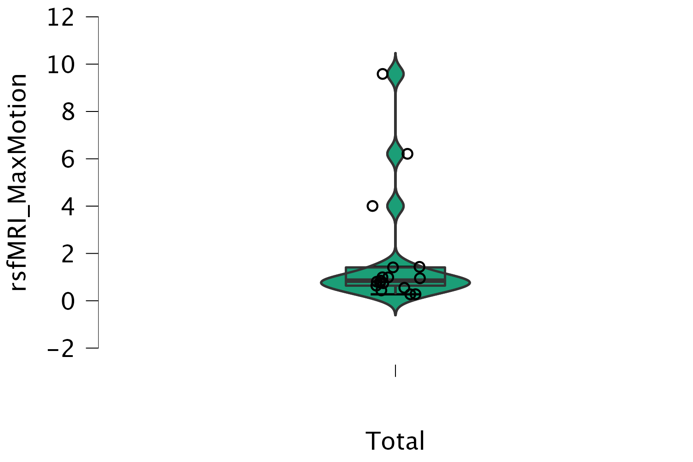

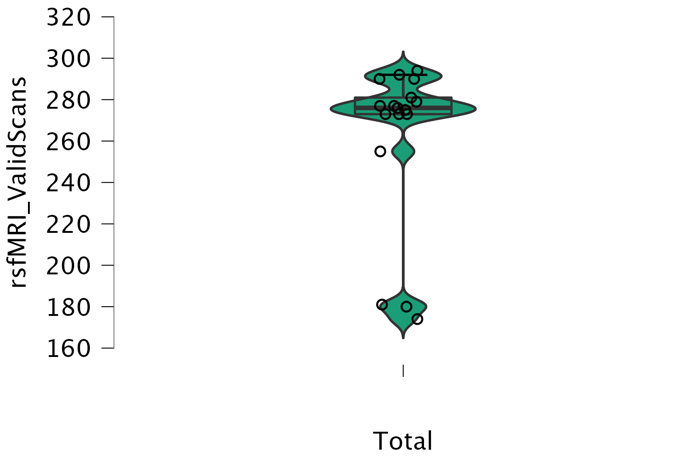
**

**Supplementary Figure 1. Resting state quality assurance.** Two Grief participants and one control were excluded from resting state analyses for excessive instances of motion (>4mm, left) and more than a third of the available timepoints were motion scrubbed (right)

**
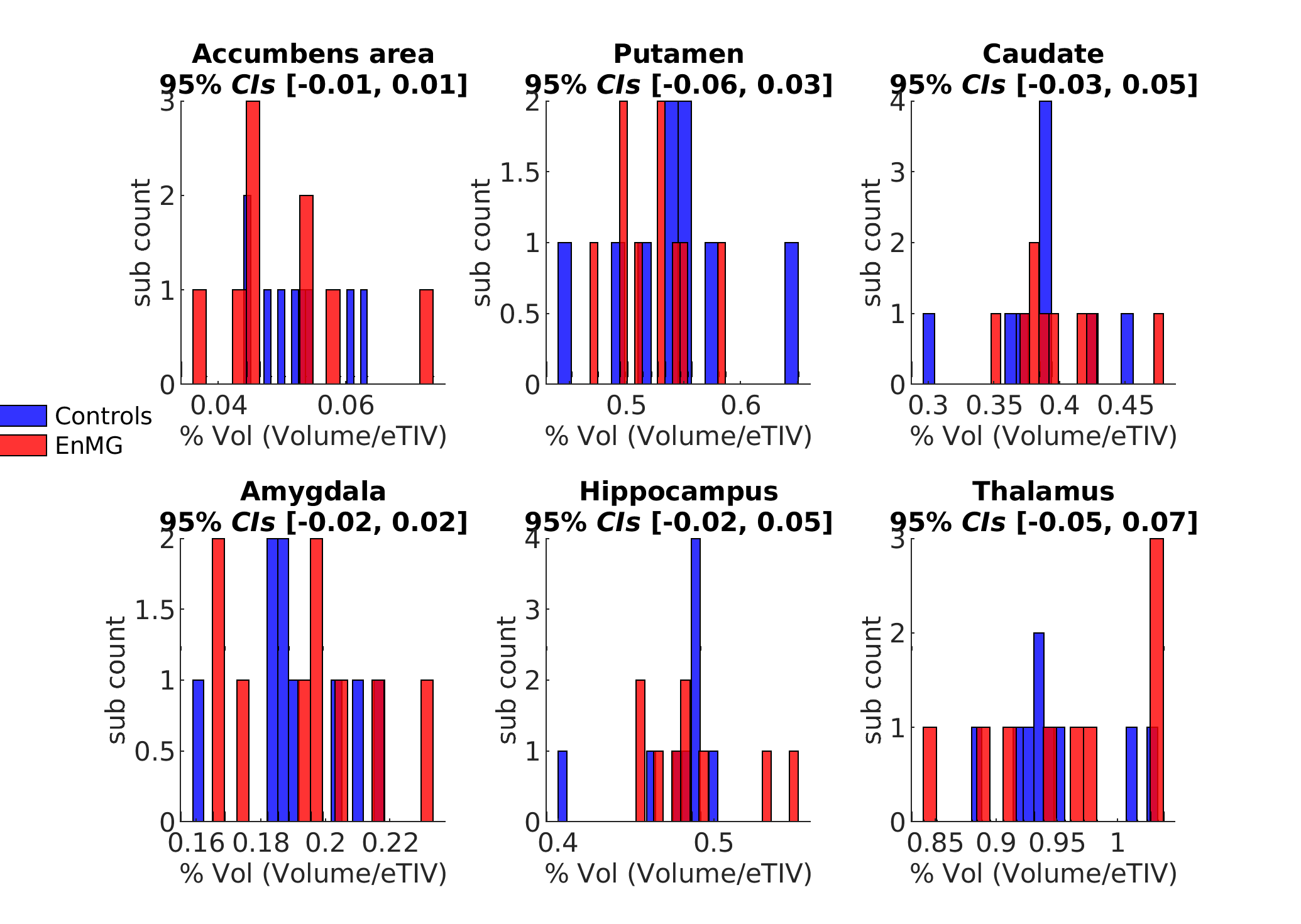
Supplementary Figure 2. Volumetric analyses.** Histograms of volumetrics in regions of interest by group. Volume measured as % (volume divided by estimated total intracranial volume). Confidence intervals for independent samples t-tests (unequal variance assumed).


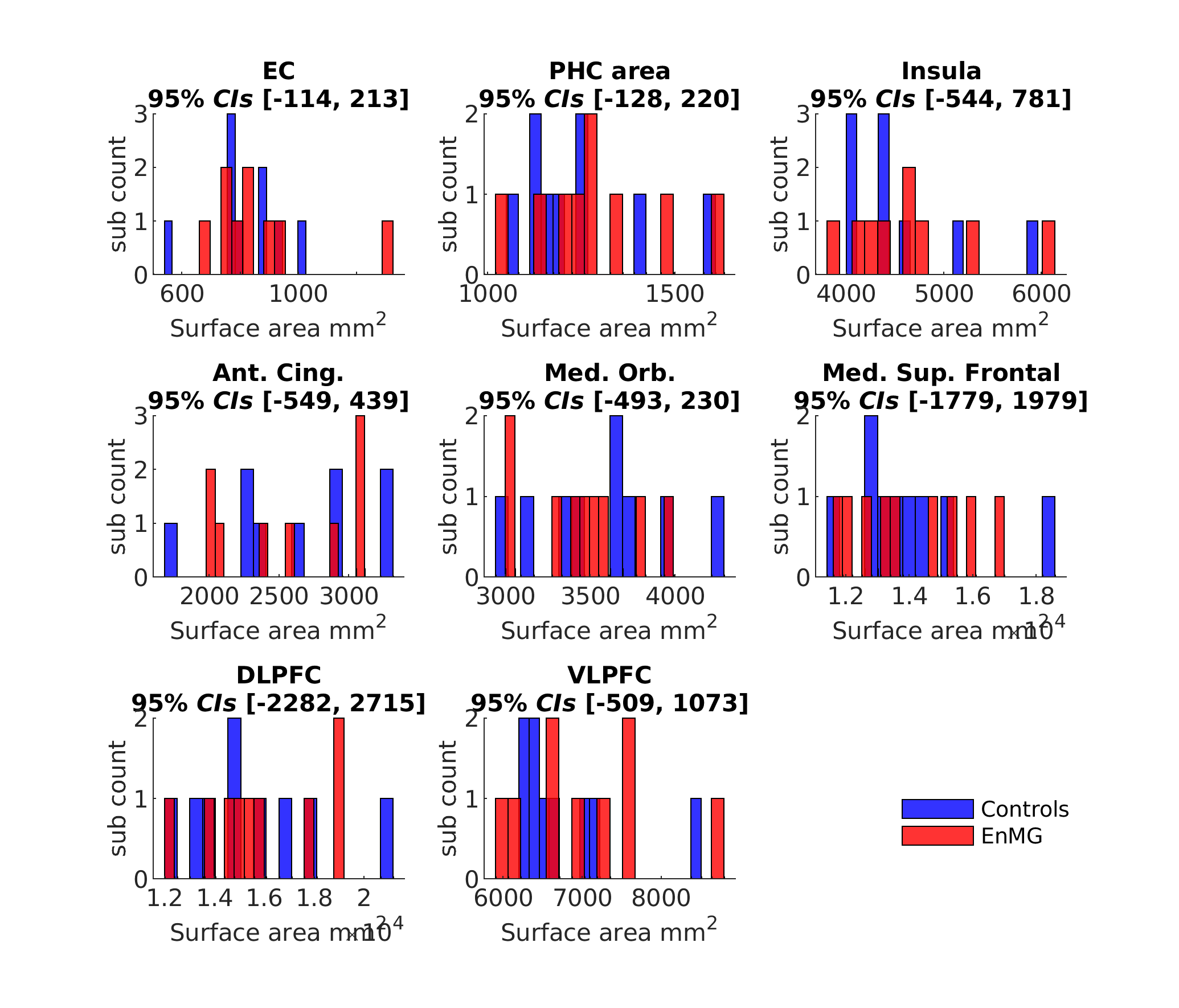


**Supplementary Figure 3.** Surface area analyses. Histograms of surface areas for regions of interest by group. Volume measured as % (volume divided by estimated total intracranial volume). Confidence intervals for independent samples t-tests (unequal variance assumed). Ant=anterior, Cing=cingulate, DLPFC=Dorso-lateral prefrontal cortex, EC=entorhinal cortex, Med=medial, Orb=orbital frontal cortex, PHC=parahippocampal cortex, VLPFC=Ventro-lateral prefrontal cortex.


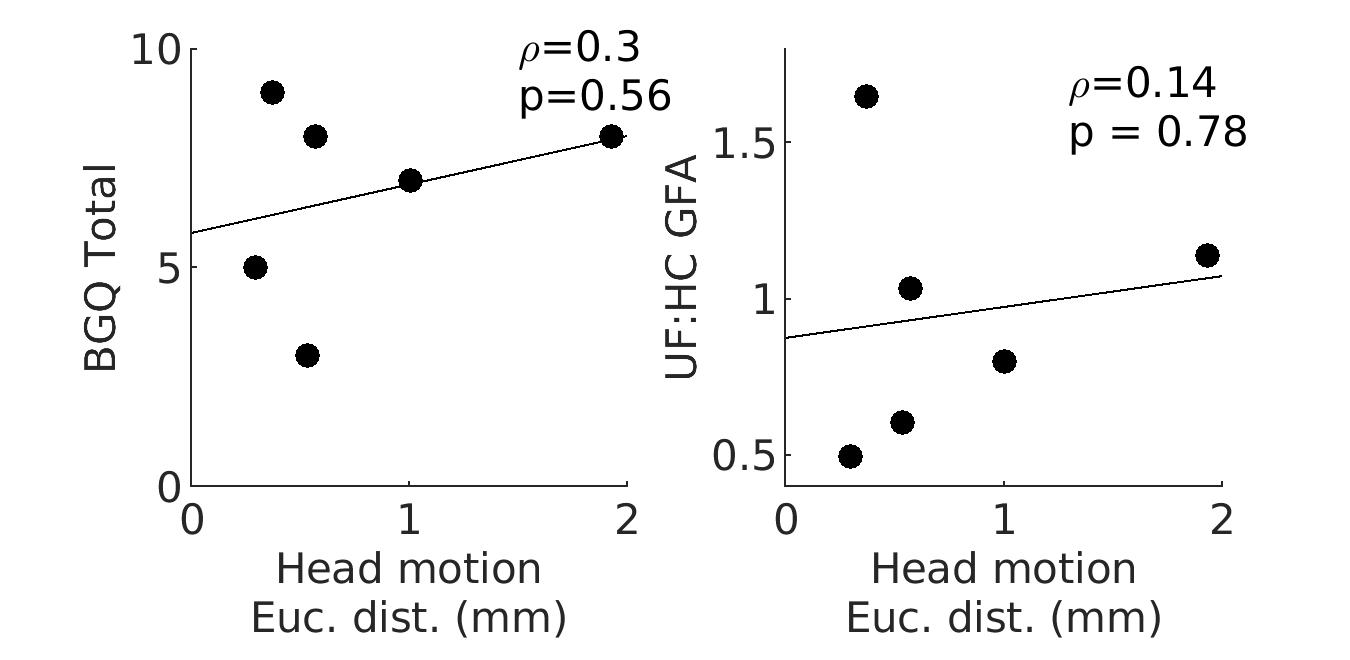


**Supplementary Figure 4.** Follow-up analysis to test if UF:HC GFA or BGQ scores tracked with DTI head motion.

**
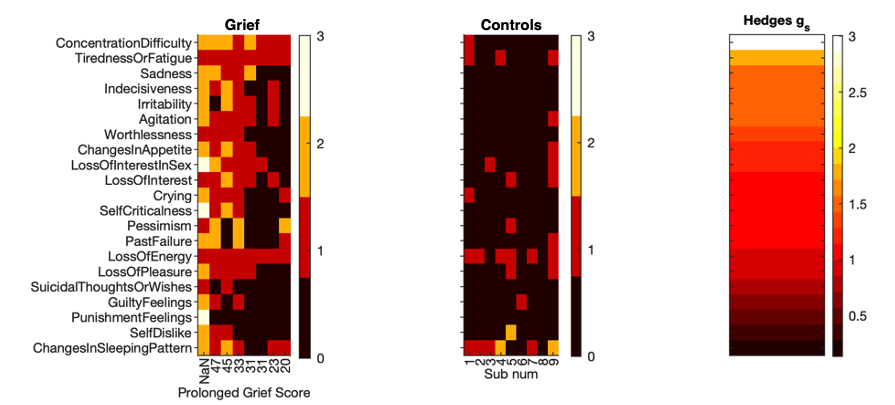
Supplementary Figure 5.** Item analysis of the BDI-II items used to probe cognitive, affective, and somatic symptoms in the Grief group (left) and Controls (right) by sorting by the row items based on the Hedges g effect size of group differences and the columns sorted by subjective grief score (PG13-Total).
